# Cholera and diarrheal diseases in Cuamba District, Niassa Province, Mozambique: Systematic healthcare facility-based surveillance strengthening, characteristics of suspected cholera and diarrheal patients, and incidence of diarrheal diseases

**DOI:** 10.1371/journal.pntd.0011843

**Published:** 2024-04-30

**Authors:** Cynthia Semá Baltazar, José Paulo Langa, Liliana Dengo Baloi, Jucunu J. Elias Chitio, José Alberto Manuel, Ramos B. J. Mboane, Sadate Assane, Alide Omar, Mariana Manso, Igor Capitine, Naira Luiz, David Mukasa, Geun Hyeog Jang, Ju Yeon Park, Florian Marks, Ramzi Mraidi, Gi Deok Pak, Deok Ryun Kim, Se Eun Park

**Affiliations:** 1 National Institute of Health, Maputo, Mozambique; 2 Provincial Health Directorate, Lichinga City, Niassa Province, Mozambique; 3 District Health Directorate, Cuamba City, Cuamba District, Niassa Province, Mozambique; 4 Clinical, Assessment, Regulatory, Evaluation (CARE) Unit, International Vaccine Institute, Seoul, Republic of Korea; 5 Biostatistics and Data Management (BDM) Department, International Vaccine Institute, Seoul, Republic of Korea; 6 Epidemiology, Public Health, Impact (EPIC) Unit, International Vaccine Institute, Seoul, Republic of Korea; 7 Department of Medicine, University of Cambridge, Cambridge, United Kingdom; 8 Yonsei University Graduate School of Public Health, Seoul, Republic of Korea; Mahidol Univ, Fac Trop Med, THAILAND

## Abstract

**Background:**

Mozambique is one of the countries in Africa that is continuously at risk of cholera outbreaks due to poor sanitation, hygiene, and limited access to potable water in some districts. The Mozambique Cholera Prevention and Surveillance (MOCA) project was implemented in Cuamba District, Niassa Province to prevent and control cholera outbreaks through a preemptive cholera vaccination, strengthened surveillance system for cholera and diarrheal diseases, and better understanding of cholera-related healthcare seeking behavior of local populations, which may further guide the national cholera control and prevention strategies. This article presents the surveillance component of the MOCA project.

**Methodology/Principal findings:**

A prospective healthcare facility (HCF)-based surveillance of cholera and diarrheal disease was conducted in six HCFs in the District of Cuamba from March 2019 to December 2020. A systematic surveillance procedure has been put in place with capacity building in selected sentinel HCFs and a basic microbiology laboratory established on-site. Patients presenting with suspected cholera or other diarrheal symptoms were eligible for enrollment. Clinical data and rectal swab samples were collected for laboratory confirmation of *Vibrio Cholerae* and other pathogens. A total of 419 eligible patients from six HCFs were enrolled. The median age was 19.8 years with a similar age distribution between sentinel sites. The majority were patients who exhibited diarrhea symptoms not suspected of cholera (88.8%; n = 410). Among those, 59.2% (210/397) were female and 59.9% (235/392) were 15 years and above. There were 2 cholera cases, coming outside of the catchment area. The incidence of diarrheal diseases ranged from 40–103 per 100,000 population. No *Vibrio cholerae* was isolated among surveillance catchment population and *Escherichia coli spp*. (82/277; 29.6%) was the most common pathogen isolated.

**Conclusion/Significance:**

Efforts were made to strengthen the systematic surveillance of suspected cholera with standardised patient screening, enrolment, and diagnostics. The first basic microbiology laboratory in Niassa Province established in Cuamba District under the MOCA project needs to be integrated into the national network of laboratories for sustainability. No reports of laboratory confirmed cholera cases from the surveillance catchment area may be highly related to the pre-emptive oral cholera vaccine (OCV) mass vaccination campaign conducted in 2018 and the use of drugs by local populations prior to visiting the sentinel HCFs. Continued systematic cholera surveillance is needed to closely monitor the cholera endemicity and epidemics, and further evaluate the long-term impact of this vaccination. High incidence of diarrheal illnesses needs to be addressed with improved water, sanitation, and hygiene (WaSH) conditions in Cuamba District. Efforts integrated with the prioritization of prevention measures are fundamental for the control of cholera in the country.

## Author summary

Mozambique faces an ongoing risk of cholera outbreaks, due to factors such as poor sanitation and limited access to clean and potable water. To address this challenge, the Mozambique Cholera Prevention and Surveillance (MOCA) project was launched in one of the high-risk areas, Cuamba District, Niassa Province, in 2018. This initiative was an integral part of Mozambique’s efforts to develop a comprehensive National Cholera Plan, aiming to bolster cholera and diarrheal disease surveillance and inform national prevention strategies. Under the MOCA project, we conducted a preemptive oral cholera vaccine (OCV) mass vaccination campaign in 2018 and implemented a healthcare facility-based surveillance for cholera and diarrheal diseases in six healthcare facilities in Cuamba District. A systematic cholera surveillance system was put in place in the study sentinel sites with standardised patient screening, enrolment, and diagnostic procedures. The first basic microbiology laboratory in Niassa province was established in Cuamba District in 2018 with funding and technical support made available under this MOCA project. A significant portion of patients in our surveillance system exhibited diarrheal symptoms unrelated to cholera, with a notable representation of females and individuals aged 15 years and older during the surveillance period in 2019–2020. No suspected cholera patients enrolled was laboratory-confirmed with *Vibrio cholerae*, which may be highly related to the cholera vaccination campaign performed in 2018 and the local populations’ tendency on the use of drugs before seeking healthcare at the HCFs. Continued surveillance is recommended to track cholera endemicity and outbreaks and also to assess the long-term impact of OCV vaccination in Cuamba District. Better infrastructure and practice of WaSH is needed to reduce the high incidence of diarrheal illnesses in the local populations. Sustainable technical and financial support is needed in the rural remote areas of Cuamba District for the continued systematic cholera and infectious disease surveillance system, including laboratory confirmation. Integrating these efforts with preventive measures is crucial for effective cholera control in Mozambique.

## Introduction

Often perceived as a disease indicative of poverty, inequity, and socio-economic underdevelopment, cholera is still a substantial public health problem in developing countries with insufficient access to safe water and proper sanitation [1–3]. Nearly 3 million cholera cases and 91,000 deaths due to cholera has been estimated every year, and almost 50% of the global cholera cases occur in the sub-Saharan Africa region [1,4,5]. Between 2000 and 2015, around 52,000 cholera deaths were reported in sub-Saharan Africa; over 83% of global cholera deaths [1]. The true burden of cholera may remain under-estimated considering the persistent challenges associated with limited systematic cholera surveillance and reporting system and lack of routine diagnosis capacity [1].

Mozambique, located in the southeastern part of sub-Saharan Africa, reports cholera cases every year with a significant cholera annual incidence, ranging from 0 to 211 per 100,000 population with periodically high case-fatality ratios (CFR) similar to estimates in the sub-Saharan region of 1.6% from 2010 to 2020 [6]. The country has a population of over 30 million [7] and shares borders with Malawi, Zimbabwe, and Tanzania, where cross-border cholera transmissions have been reported [8]. Since the first cholera report in Mozambique in 1970, cholera has been one of the major public health concerns in the country for decades [9]. Since 1989, Mozambique has experienced several large cholera outbreaks with over 7,000 cases reported between September 2015 and March 2017 [10,11]. Cholera is considered an endemic disease in Mozambique with periodic epidemics almost every year with seasonal patterns [10,11]. Cholera is among the mandatory-reporting diseases identified and documented within Mozambique’s healthcare system, utilizing the framework of the World Health Organization’s Integrated Disease Surveillance and Response (IDSR) protocol [12]. In the past ten years, northern Mozambique in particular has been disproportionately affected compared to the other regions in-country, and the risk factors have been attributed to lack of adequate sanitation and environmental condition, potable water, and cultural and hygiene practices [9–11]. In 2015, Mozambique was heavily affected by an epidemic with total 7,073 cholera cases and 54 cholera deaths in five Provinces (Tete, Nampula, Niassa, Zambézia, and Sofala) [11]. Mozambique is prone to natural disasters such as cyclones and regular flooding along its main rivers in the Zambezi and Limpopo river basins, which has often resulted in the increase of infectious water-borne disease outbreaks, particularly cholera [13–15]. In 2019, the country was devastated by two consecutive tropical cyclones Idai and Kenneth that hit the central and northern parts of Mozambique, respectively. After cyclone Idai hit the country, a cholera outbreak was declared in Sofala Province with a total of 6,768 suspected cholera cases and 8 deaths associated with cholera [13]. The cyclone Kenneth resulted in 254 suspected cholera cases with no cholera deaths in Cabo Delgado Province [14].

Persistent cholera outbreaks across the country have been associated with multiple risk factors, including rapid population growth of approximately 3% every year [16], large-scale wartime population displacement during the health emergencies caused by weather events, and gaps in water, sanitation and hygiene (WaSH) infrastructure and hygiene practice, despite efforts to improve provision of sanitation system and water supply [9,11,17]. The country has one of the lowest levels of safe water consumption in the world; Mozambique ranks 128^th^ and 119^th^ out of 135 countries concerning access to improved source of water and sanitation respectively (6). While WaSH improvement is a long-term sustainable solution to ultimately control and prevent cholera and other water-borne infectious diseases, the administration of oral cholera vaccines (OCV) is an effective public health intervention for emergency cholera outbreak control. The World Health Organization (WHO) position paper in 2017 has recommended the use of OCV in outbreak and endemic settings, combined with other prevention and control strategies [18,19]. Further, countries need to strengthen the national surveillance system as a key strategy to improve the accuracy of cholera case detection and reporting, timely monitoring of cholera outbreaks in endemic or non-endemic areas and identifying hotspots.

Based on the public health prioritization exercise for the development of cholera conducted by the Ministry of Health (MOH) of Mozambique in partnership with the WHO in 2016, as part of a comprehensive strategy for cholera prevention and control in endemic setting, the District of Cuamba and the City of Nampula were assessed as one of the high priority areas for controlling and preventing cholera. Cuamba District is located within Niassa Province adjacent to Nampula and was designated as endemic for cholera with periodic outbreaks including the cholera epidemic in 2015. Over 200 suspected cholera and 2,000 diarrheal cases were reported almost every year, with an exception of 2014 and 2016 [10]. The Mozambique Cholera Prevention and Surveillance (MOCA) project, jointly led by the Mozambique National Institute of Health (INS), Niassa Health Directorate, and the International Vaccine Institute (IVI), aimed to prevent and control cholera outbreaks through: a preemptive oral cholera vaccine (OCV) mass vaccination campaign in this cholera endemic and hotspot Cuamba District in 2018; followed by local public health system strengthening in cholera and diarrheal disease surveillance in 2019–2020; and investigate the disease incidence and clinical characteristics. Here, we present the results of the two-year surveillance of cholera and diarrheal diseases, including frequencies and incidences of cholera and other diarrheal diseases, etiologies of diarrheal cases commonly detected in the surveillance catchment population, and clinical characteristics of suspected cholera and diarrheal patients. The result of our OCV mass vaccination campaign including the coverage rates has been published and publicly available [20])

## Methods

### Ethical consideration

The study protocol was approved by the Institutional Ethics Committee of the National Institute of Health (CIBS:78/CIBS/2017), Mozambican National Bioethics Committee for Health (CNBS: 116/CNBS/19) and the IVI Institutional Review Board (IRB: 2018–001). A written informed consent was obtained from all eligible patients. For minors below 18 years, assent form was obtained and informed consent from parents or guardians or legally authorized persons. The study adhered to the national guidelines for cholera treatment and care, ensuring that the well-being and confidentiality of all participants were upheld throughout the study.

### Study site and population

The Cuamba District is located in Niassa Province in northern Mozambique. With a population size estimation of around 264,572, Cuamba district comprises 36 neighborhoods known locally as “bairros and povoados” [7]. The healthcare facilities (HCFs) in Cuamba District have been assessed jointly by the IVI and INS study team between 2017–2018. Based on these site assessments and review of epidemiological data of cholera cases in Cuamba District, a total of six HCFs (Cuamba District Hospital, Cuamba District Health Facility, Namutimbua Health Center, Tetereane Health Center, Adine III Health Center, and Mujaua Health Center) with concentrated cholera cases in the past five years have been selected for the implementation of prospective sentinel HCF-based cholera and diarrheal disease surveillance.

### Case definition

All residents of all ages living in the surveillance catchment areas within Cuamba District, who presented to the sentinel HCFs for the MOCA project were eligible for screening of the following study inclusion criteria: clinically suspected cholera *OR* diarrhea. Case definitions adhered to WHO guidelines, where suspected cholera was defined as those with acute watery diarrhea (lasts several hours or days) or severe dehydration, OR acute watery diarrhea with or without vomiting [21]. Diarrhea cases included patients with persistent diarrhea (lasts 14 days or longer), acute bloody diarrhea (also called dysentery), and acute watery diarrhea (lasts several hours or days) that included cholera.

### Surveillance strengthening and data and sample collection

Under the MOCA project, the healthcare facility-based cholera and diarrheal disease surveillance system was strengthened. For a systematic screening and testing of clinically suspected cholera and diarrheal patients, standard operating procedures (SOPs) for screening and enrolment of eligible patients, sample collection, handling, and diagnostics using cholera rapid diagnostics test (RDT) and culture method were put in place. Trainings and refresher trainings were conducted for healthcare professionals stationed at the surveillance sentinel HCFs and laboratory technicians. The MOCA project also supported the establishment of the first basic microbiology laboratory in the Niassa province. This laboratory was built within the compound of the secondary referral hospital in Cuamba District.

All patients who met the inclusion criteria and consented to participate in the study (written informed consent) were enrolled. In cases where participants could not provide a written signature due to illiteracy, they provided verbal consent and used a finger stamp in the presence of a witness. Clinical information was collected in patient enrolment form (PEF) that included basic metadata such as sex, age, medical history, symptoms, and clinical diagnosis. Rectal swab samples were collected for cholera rapid diagnostic tests (RDT) and laboratory diagnosis. Rectal swabs were collected in transport medium and sent from each sentinel HCFs to the established Cuamba Microbiology Laboratory for primary culture and reconfirmation at the INS laboratory in Maputo. Laboratory results were captured in laboratory form (LF) that included the culture results and antimicrobial susceptibility.

### Laboratory diagnostics

Rectal swab samples were collected and cultured using the TCBS (thiosulfate-citrate-bile-salts-sucrose), XLD (xylose-lysine-deoxycholate) and SS (*Salmonella/Shigella*) agar for detection of *Vibrio spp*. and other diarrheal pathogens. Specimens collected were first sent to the Cuamba Microbiology Laboratory that has been established within the Cuamba District Hospital compound under the MOCA project for culture on a daily basis, using Cary-Blair medium to maintain the viability of microorganisms. Then, all samples with positive growth were transported to Lichinga Provincial Laboratory in a container with transportation medium (Cary-Blair), and subsequently to the Microbiology Reference Laboratory at INS located in Marracuene, Maputo Province for re-confirmation of the isolates and quality control. An antimicrobial susceptibility test was also performed on the isolates yielded through rectal swab culture. For patients suspected of having cholera, a rapid diagnostic test (RDT: Crystal VC test kit, 16IC101-10, Span Diagnostics, Surat, India) was conducted for patient care and adequate treatment at HCFs. The RDT is able to detect *V*. *cholerae* O1 and O139 [22–24].

### Data analysis

The paper-based clinical data collected were entered into the desk-top based database system, IVI Data Center (IDC), developed by IVI in-house based on an executable JAR file running on local computers. The paper-based lab data were entered into an excel-based database. The adjusted incidences of diarrheal diseases were estimated per 100,000 person years-observation (PYO) with surveillance catchment population adjusted by healthcare seeking behaviour. We used clinical data collected in patient enrolment form during the surveillance period to ascertain the number of patients with clinically suspected cholera or diarrhea. The clinical characteristics of suspected cholera and diarrheal patients enrolled, and laboratory confirmed organisms associated with enrolled patients are presented in frequencies and proportions. All the analyses were performed using SAS ver. 9.4 (SAS Institute Inc., Cary, NC).

## Results

### Eligible patient enrollment per sentinel healthcare facilities

From March 2019 to December 2020, a total of 7,593 patients were screened for the study eligibility at six sentinel healthcare facilities: Cuamba District Hospital, Cuamba District Health Center, Namutimbua Health Center, Tipo II–Tetereane Health Center, Adine III Health Center, and Mujaua Health Center (Table 1). Among them, 530 patients were eligible for enrolment according to the study inclusion criteria, of which 419 eligible patients were enrolled based on the informed consent. More patients were enrolled from Adine III Health Center (123/419; 29.4%) and Namutimbua Health Center (104/419; 24.8%) than the other sentinel health centers. The median age of all enrolled patients was 19.8 years (interquartile range (IQR) 2.1–28.0) with most sentinel health centers showing similar age distributions. Notably, the median age of enrolled patients in Namutimbua and Mujua Health Centers were much younger; 7.1 (IQR 1.3–24.2) years and 1.7 (IQR 0.9–3.4) years, respectively. The majority of enrolled patients exhibited non-cholera diarrheal illness while nine clinically suspected cholera patients (9/419; 2.2%) were enrolled. Total 49 enrolled patients were admitted to inpatient wards, largely at the Cuamba District Hospital (38/52; 73.1%). Rectal swab samples were collected from around 69.9% (293/419) of total enrolled patients.

**Table 1:** MOCA surveillance enrolment.

Study site			Cuamba District				
Site setting			Semi-Rural/Semi-urban				
Population^1^			265,425 (131,808 male; 133,617 female)				
Surveillance period (start-end)			21 March 2019–31 December 2020				
Surveillance sentinel network (HCFs)	n	Cuamba District Hospital (Secondary/ Referral)	Cuamba District Health Center (Primary)	Namutimbua Health Center (Primary)	Tipo II–Tetereane Health Center (Primary)	Adine III Health Center (Primary)	Mujaua Health Center (Primary)
**Total patients screened**	7,593						
**Total patients eligible**	530						
**Patients enrolled, N = 419 &** **n per HCF (n/N (%)**	419	54/419 (12.9)	64/419 (15.3)	104/419 (24.8)	53/419 (12.7)	123/419 (29.4)	21/419 (5.0)
**Median age, years (25–75%), N = 400**	19.8 (2.1–28.0)	25.0 (5.4–37.7)	22.2 (17.0–29.0)	7.1 (1.3–24.2)	25.8 (19.7–38.0)	18.0 (2.0–26.0)	1.7 (0.9–3.4)
**Proportion female, n (% of enrolled)**	213/406 (52.5)	30/51 (58.8)	39/63 (61.9)	44/101 (43.6)	27 /53(50.9)	62/118 (52.5)	11/20 (55.0)
**Suspected cholera patients, n (% of enrolled)**	9/419 (2.2)	4/54 (7.4)	2/64 (3.1)	0/104 (0)	0/53 (0)	2/123 (1.6)	1/21 (4.8)
**Diarrheal patients not suspected of cholera, n (% of enrolled)**	410/419 (97.9)	50/54 (92.6)	62/64 (96.9)	104/104 (100.0)	53/53 (100.0)	121/123 (98.4)	20/21 (95.2)
**Inpatients, n (% of enrolled)**	49/391 (12.5)	38/52 (73.1)	2/52 (3.9)	2/99 (2.0)	2/52 (3.9)	4/120 (3.3)	1/16 (6.3)
**Rectal swab samples collected, n (% of enrolled)**	293/419 (69.9)	33/54(61.1)	49/64 (76.6)	72/104 (69.2)	41/53(77.4)	86/123 (69.9)	12/21 (57.1)

^1^Catchment population data source: 2021 national census projection.

### Characteristics of suspected cholera and diarrheal patients enrolled

Among the nine enrolled suspected cholera patients, 3 (33.3%) were female and nearly all of them (87.5%) were aged 15 years and above (Table 2). For patients with non-cholera suspected diarrheal illness, around 53% (210/397) were female and nearly 60% (235/392) were aged 15 years and above, followed by younger children aged below 2 years that constituted over 23% (91/392). Notably, 4 out of 9 suspected cholera patients and 186 out of 410 non-cholera suspected diarrheal patients confirmed having received the OCV; for suspected cholera patients, 2 cases with single-dose (SD) and another 2 cases with two-doses (2D), and 52 (28% of 186) and 123 (66% of 186) non-cholera suspected diarrheal patients with SD and 2D respectively. Total 7 enrolled patients (2 suspected cholera and 5 non-cholera suspected diarrheal patients) informed history of cholera, defined as having had cholera in the past before visiting the study HCFs during the study surveillance period.

The majority of patients enrolled reported symptoms of diarrhea on the day of and from one to two days prior to HCF visit (75% among suspected cholera and 98.9% among non-cholera suspected diarrheal patients). Most common symptoms exhibited in cholera suspected patients were abdominal cramp (4/9; 44%), vomiting (4/9; 44%) and signs of dehydration such as thirst (3/9; 33%), sunken eyes (2/7; 29%), moist extremities (2/9; 22%), decreased skin elasticity (1/9; 11%), lowered consciousness (1/9; 11%), and weak pulse (1/9; 11%). Similar characteristics were reported for the non-cholera suspected diarrheal patients enrolled, including sunken eyes (218/372; 59%), abdominal pain (205/389; 53%), thirst (200/394; 51%) and vomiting (101/399; 25%).

Around 77% (19% of 404) of enrolled patients had pre-use of drugs or rehydration therapy prior to visiting the HCFs; 1 suspected cholera patient and 76 non-cholera suspected diarrheal patients. In both groups, the majority (305/392; 78%) received rehydration therapy during HCF visit; 6 (67% of 9) cholera suspected patients and 299 (78% of 383) non-cholera suspected diarrheal patients. Most rehydration therapy was oral rehydration solution (ORS, 257/305; 84%) but some patients (47/305; 15%) received intravenous fluids (IV fluids). Around 13% (49/391) of enrolled patients were hospitalized; 1 female suspected cholera patient aged above 15 years (1/9; 11%), and 48 non-cholera suspected diarrheal patients (48/382; 13%) that included 53% (24/45) female and 66% (29/44) aged above 15 years, followed by 23% (10/44) aged below 2 years and 11% (5/44) aged between 5 and 14 years (Table 2).

**Table 2:** Characteristics of suspected cholera and diarrheal patients enrolled.

	Suspected cholera N = 9	Diarrhea not suspected of cholera N = 410
**Female**	3/9 (33.3)	210/397 (52.9)
**Age, n (%)**		
** <2 years**	0/8 (0)	91/392 (23.2)
** 2<5 years**	0/8 (0)	34/392 (8.7)
** 5<15 years**	1/8 (12.5)	32/392/ (8.2)
** 15 years and above**	7/8 (87.5)	235/392 (59.9)
**OCV received, n (%)**		
Don’t Know	0/4 (0)	11/186 (5.9)
1 dose	2/4 (50.0)	52/186 (28.0)
2 doses	2/4 (50.0)	123/186 (66.1)
Female, n (%)		
Pregnant when receiving OCV doses	0/3 (0)	6/210 (2.9)
**History of cholera, n (%)**	2/9 (22.2)	5/399 (1.3)
**Enrolled patients with watery stools n (%)**		
** Watery Stools Today**	6/7 (85.7)	331/333 (97.9)
** Watery Stools Yesterday**	7/8 (87.5)	360/364 (98.9)
** Watery Stools 2 days ago**	3/4 (75.0)	100/104 (96.15)
Rice water stool	0/9 (0)	40/410 (9.8)
Bloody stool	1/9 (11.1)	33/410 (8.1)
Slimy stool	6/9 (66.7)	210/410 (51.2)
**Other clinical characteristics, n (%)**		
Signs of dehydration		
Sunken eyes	2/7 (28.6)	218/372 (58.6)
Thirsty	3/9 (33.3)	200/394 (50.8)
Decreased skin elasticity	1/9 (11.1)	64/403 (15.9)
Cold or moist extremities	2/9 (22.2)	30/401 (7.5)
Lowered consciousness	1/9 (11.1)	14/393 (3.6)
Low urine output	0/9 (0)	52/398 (13.1)
Weak pulse	1/9 (11.1)	25/397 (6.3)
Restlessness	0/9 (0)	48/392 (12.2)
Vomiting	4/9 (44.4)	101/399 (25.3)
Abdominal cramps	4/9 (44.4)	205/389 (52.7)
Rapid or difficulty breathing	0/8 (0)	49/375 (13.1)
**Pre-use of drugs or rehydration therapy prior to visit to HCFs, n (%)**	1/7 (14.3)	76/397 (19.1)
**Case Management, n (%)**		
** Received rehydration therapy during HCF visit**	6/9 (66.7)	299/383(78.1)
Oral Rehydration Solution (ORS)	5/6 (83.3)	252/299 (84.3)
** Intravenous (IV) fluids**	1/6 (16.7)	46/299 (15.4)
** Drugs prescribed during HCF visit**	6/9 (66.7)	365/410 (89.0)
** Hospitalization, n (%)**	1/9 (11.1)	48/382 (12.6)
** Female**	1/1 (100)	24/45 (53.3)
** Age among all hospitalized, n (%)**		
** <2 years**	0/1 (0)	10/44 (22.7)
** 2<5 years**	0/1(0)	0/44(0)
** 5<15 years**	0/1 (0)	5/44 (11.4)
** 15 years and above**	1/1 (100)	29/44 (65.9)

### Laboratory findings

Of total 419 enrolled patients, 293 rectal swab samples were collected including 282 samples with proper study IDs (5 suspected cholera cases and 277 non-suspected cholera diarrheal cases) and laboratory culture results (Table 3). Out of samples cultured, 282 exhibited positive growth. Most commonly yielded isolates were *E*. *coli* (83/282; 29%) followed by *Proteus mirabilis* (26/282; 9%) and *Providencia spp*. (26/282; 9%). There were two *V*. *cholerae* isolates detected, but the cases were local soldiers passing through Cuamba District, and thus outside the study surveillance catchment area. One *V*. *fluvialis* was detected from rectal swab sample collected from non-cholera suspected diarrheal patients.

**Table 3:** Laboratory confirmation of organisms associated with enrolled patients.

	Suspected cholera (N = 9)	Diarrhea not suspected of cholera (N = 410)
**Culture result** ^**1**^		
** Positive growth**	5	277
**Real pathogens**		
** *Vibrio cholerae*** ^**2**^	0/5 (0)	2/277 (0.7)
** *Vibrio fluvialis & Enterobacter cloacae***	0/5 (0)	1/277 (0.4)
** *Acinetobacter spp*.**	0/5 (0)	4/277 (1.4)
** *Citrobacter spp*.**	0/5 (0)	17/277 (6.1)
** *Enterobacter cloacae***	0/5 (0)	1/277 (0.4)
** *Enterobacter spp*.**	0/5 (0)	22/277 (7.9)
** *Escherichia coli spp*.**	1/5 (20.0)	82/277 (29.6)
** *Inactive E*. *coli***	0/5 (0)	2/277 (0.7)
** *Klebsiella oxytoca***	0/5 (0)	3/277 (1.1)
** *Klebsiella pneumoniae***	0/5 (0)	33/277 (11.9)
** *Klebsiella spp*.**	0/5 (0)	2/277 (0.7)
** *Morganella morganii spp*.**	0/5 (0)	15/277 (5.4)
** *Non Fermentator***	0/5 (0)	12/277 (4.3)
** *Oxidase positive***	0/5 (0)	6/277 (2.2)
** *Proteus mirabilis***	1/5 (20.0)	25/277 (9.0)
** *Proteus vulgaris***	1/5 (20.0)	8/277 (2.9)
** *Providencia spp*.**	2/5 (40.0)	24/277 (8.7)
** *Pseudomonas spp*.**	0/5 (0)	2/277 (0.7)
** *Serratia spp*.**	0/5 (0)	12/277 (4.3)

^1^ 293 samples collected out 419 enrolled patients, which included 282 samples with proper study IDs (5 suspected cholera cases plus 277 other diarrheal cases) and lab culture results and used for this analysis.

^2^ The two *V*. *cholerae* cases detected were non-residents of Cuamba District, and thus outside the study catchment area with non-cholera suspected diarrheal symptoms.

### Adjusted incidence of diarrheal diseases

Overall, the adjusted incidence of diarrheal diseases in the surveillance areas in Cuamba District was 106 per 100,000 person-year-observation (PYO) (Table 4). Children under two years of age exhibited the highest diarrheal disease incidence (754 per 100,000 PYO). Children aged between two to four years also showed high burden of diarrheal diseases (71 per 100,000 PYO). Notably, people aged 15 years and above also had a significant burden of diarrhea with the adjusted incidence estimate of 103 per 100,000 PYO.

**Table 4:** Adjusted incidence estimates of diarrheal diseases.

Age (years)	Proportion of population seeking healthcare at study HCFs *in diarrhea*^1^	PYO Estimation			Adjusted incidence of diarrheal diseases	
		Catchment population^2^	Catchment population adjusted by health seeking behavior	PYO^3^	Crude cases of all diarrheal cases^4^	Adjusted incidence per 100,000 PYO (95% CI)
0–1 years	52%	10,402	5,409	12,073	91	754 (614–926)
2–4 years	58%	37,098	21,517	48,018	34	71 (51–99)
5–14 years	51%	71,965	36,702	81,907	33	40 (29–57)
<15 years		119,465	63,628	141,998	158	111 (95–130)
≥15 years	72%	145,960	105,091	234,529	242	103 (91–117)
All ages		265,425	168,719	376,527	400	106 (96–117)

^1^ Proportion of population seeking healthcare at study HCFs in diarrhea is based on the healthcare utilization survey conducted in the MOCA surveillance catchment area in 2019.

^2^ Catchment population data source: 2021 national census projection.

^3^ Person-years-observation (PYO) was used as a denominator to estimate the adjusted incidence rates of diarrheal illnesses presented in this table. The PYO was calculated using the surveillance catchment population data adjusted by health seeking behaviour associated with diarrheal illnesses and the duration of surveillance period (March 2019 to December 2020).

^4^ Suspected cholera cases not included.

## Discussion

In Mozambique despite the trend of diarrheal diseases declined in the last ten years, it is still one of the major public health issues [10,11] and the fourth leading causes of morbidity and mortality in children under five years of age [25]. In our sentinel surveillance sites, HCFs showed a different pattern of diarrheal cases (who were not suspected of cholera), with the enrollment range of 5–29.4%. Namutimbua HCF had more eligible patients enrolled and showed lower median age of the enrolled patients (median age 7.1 years). This finding has important implications in considering adequate public health interventions for effective reduction of diarrheal illnesses in children. Notably, we have also found a high incidence of diarrheal diseases in older children and adults aged 15 years and above, indicative of the overall poor WaSH infrastructure and practice in local populations living in Cuamba District. Most hospitalized patients occurred in Cuamba District Hospital (73.1%), which may be due to the fact that this is the main district health facility (secondary and referral hospital) with capacity to provide healthcare services for inpatients.

No laboratory confirmed cholera cases were reported during the implementation of our enhanced surveillance system in Cuamba from March 2019 to December 2020; the period of 1–2 years following the two-dose pre-emptive OCV mass vaccination campaign conducted in 2018 under the MOCA project [20]. The two cases of *Vibrio cholera* and the one *Vibrio fluvialis* identified were from individuals residing in other parts of Nampula Province, which is known to be one of the cholera endemic regions in Mozambique [11,26]. Our age distribution analysis revealed that the majority of suspected cholera cases occurred mostly in men aged above 15 years, though the laboratory diagnostics result was negative for *V*. *Cholerae* but positive for non-cholera diarrheal pathogens. In a similar surveillance system strengthening activity conducted in Mozambique during 2011, the majority of suspected cholera cases (53%) were male, with the highest proportion of suspected cases observed among men aged 20 years. Furthermore, the majority of cases occurred in individuals aged 16–25 years [11]. Our laboratory findings of acute diarrheal diseases identified mainly *E*.*coli*. Other studies conducted in the region indicate that *E*. *coli* is highly associated with moderate to severe diarrhea. Diarrhea case distribution by sex and age group showed common patterns as some other studies in Mozambique, where a high proportion of suspected cases occurred in female and adults aged over 15 years and children under 5 years [11,25,27,28]. In addition, most diarrheal cases occurred during the rainy seasons from November to January.

Nearly 20% of patients who visited our sentinel HCFs with suspected cholera or other diarrheal illnesses answered that they had pre-treatment with drugs prior to visiting the HCFs. More than 50% of the enrolled eligible patients reported having received the OCV. In 2018, a two-dose pre-emptive OCV mass vaccination campaign targeting approximately 180,000 individuals aged above one year was implemented in Cuamba District [20]. The vaccination coverage survey indicates that 60.4% (±3.4%) of the target population received full two-doses of OCV. There is circumstantial evidence that OCV and WaSH are effective and affordable interventions to prevent and control cholera [26,29,30]. The use of antibiotics prior to enrolment at our sentinel HCFs and the OCV vaccination conducted about a year prior to the surveillance period may explain the zero laboratory confirmed cholera cases in the surveillance area during 2019–2020.

Our surveillance system allowed for the measurement of diarrhea incidence. The high incidence rates of diarrheal diseases in Cuamba District affected not only children under 5 years but also the adult population, suggesting several underlying factors. Environmental conditions, such as limited access to clean water and inadequate sanitation facilities, are likely contributing to this widespread issue. The common causative pathogens identified in our surveillance implies a significant risk of waterborne transmission. This situation is further exacerbated by the lack of proper hygiene practices in the community [9,11,28]. Our incidence estimation considered the potential missed opportunities of screening and enrolling eligible patients due to the healthcare seeking behaviour of local populations. In addition, patients may not seek health advice when symptoms are mild. Enhancing the surveillance system to accurately capture all suspected cholera cases, including those not seeking medical service, may also be crucial for the better control of cholera transmission in the communities. Active community engagement and sensitization is necessary for early detection and referral of clinically suspected cholera cases to the adequate HCFs or/and cholera treatment centers.

Although our study provides valuable information about diarrhea surveillance in Cuamba District, there are some limitations. Our diagnostic procedures relied upon culture results, which can be influenced by various factors including collection, transport and storage conditions considering that Cuamba is a remote area. Despite these limitations, our surveillance system yielded high quality results. For this, it’s important to acknowledge the pivotal role our Cuamba Microbiology Laboratory played in ensuring the reliability and accuracy of culture results, overcoming the logistical challenges inherent in remote areas. By integrating the Cuamba Microbiology Laboratory into the national laboratory network, we have been able to adopt a more coordinated approach to disease surveillance and management. This integration not only enhances our capacity for early detection and timely response to outbreaks but also ensures consistency and standardization of diagnostic procedures across the network. Furthermore, we believe it’s important to highlight that our two-dose pre-emptive OCV campaign likely played an important role in controlling cholera in Cuamba District in the subsequent years post-vaccination. However, to comprehensively assess the long-term impact of the OCV vaccination campaign conducted in 2018, further investigation is needed. We recommend additional research on assessing the sustained effectiveness and impact of OCV vaccinations in similar settings and their implications for cholera control. These findings can significantly inform policy recommendations and guide public health strategies aimed at reducing the burden of cholera in remote areas like Cuamba.

## Conclusion

An enhanced and systematic cholera and diarrheal surveillance system is highly important to identify, treat, and prevent cholera and diarrheal patients in a timely manner. Continued quality surveillance is important to closely monitor cholera endemicity and outbreaks and to assess the long-term impact of OCV vaccination in Cuamba District. The WaSH infrastructure and practice need improvements to reduce the high incidence of diarrheal illnesses in both children and adult populations and better control community transmissions. Sustainable technical and financial support is needed in urban and rural remote areas of Cuamba District for the continuation of systematic cholera and infectious disease surveillance, including laboratory confirmation. This research has broader implications for public health in Mozambique. It has the potential to inform policy recommendations and guide the development of targeted public health strategies aimed at reducing the cholera burden, particularly in remote areas like Cuamba.
